# Incidence, Prognostic Factors, and Treatment Impact on Survival in Natural Killer/T-Cell Lymphoma: Population-Based Study in the United States

**DOI:** 10.2196/70129

**Published:** 2025-05-15

**Authors:** Yi Zhao, Yi Zhong, Mengqi Xiong, Linlin Huang, Xiujin Ye, Jingsong He

**Affiliations:** 1 First Affiliated Hospital Zhejiang University, No.79 Qingchun Road, Hangzhou, 310003, China, 86 057187235975; 2 Institute of Hematology, Zhejiang University, Hangzhou, China

**Keywords:** NK/T-cell lymphoma, incidence, prognostic factors, chemoradiotherapy, SEER

## Abstract

**Background:**

Natural killer/T-cell lymphoma (NKTL) is a rare malignancy of mature natural killer/T-cells, predominantly found in Asian and South/Central American populations, with limited studies conducted in Europe and the United States.

**Objective:**

The aim of this study is to present an overview of the incidence rate, demographic and clinical characteristics, treatment options, overall survival (OS), and factors influencing OS of NKTL in the United States.

**Methods:**

We used data from the Surveillance, Epidemiology, and End Results 17 database to analyze NKTL cases recorded between 2000 and 2020. In a cohort of 1162 patients with NKTL, we calculated the incidence rates and performed statistical analyses to evaluate OS, the effect of radiotherapy and chemotherapy on survival, and lymphoma-specific survival.

**Results:**

The mean annual incidence rate of NKTL in the United States was 0.067 per 100,000, with higher rates observed in men compared to women, and an increase noted with age. However, there has been no significant rise in incidence over recent years. Significant racial disparities were observed, with higher incidence rates in non-Hispanic Asian or Pacific Islanders and Hispanic people. The median survival time for patients with NKTL was 21 months, with a 5-year OS rate of 39.5%, which has shown improvement in recent years. Key independent prognostic factors impacting patient survival included age at diagnosis, clinical stage, nasal type presentation, presence of systemic symptoms, and treatment modality. Patients receiving combined radiotherapy and chemotherapy exhibited the best outcomes, with a median OS of 138 months and a 5-year OS rate of 58%. This survival benefit remained consistent even in patients with stage I/localized nasal type lymphoma, achieving a 5-year OS rate of 73.3%.

**Conclusions:**

The incidence of NKTL has remained stable in recent years. Patients with the nasal type generally experience better survival outcomes. The use of combined radiotherapy and chemotherapy appears to enhance survival, though further validation through prospective multicenter clinical trials is necessary.

## Introduction

Natural killer (NK)/T-cell lymphoma (NKTL) is an aggressive form of non-Hodgkin lymphoma that originates from mature T-cells and NK cells. It is distinct from other peripheral T-cell lymphomas, primarily affecting the nasal cavity and nasopharynx, and is closely associated with Epstein-Barr virus (EBV) infection [1]. Previously classified in the 4th and earlier editions of the *WHO Classification of Haematolymphoid Tumours* as “NK/T-cell lymphoma, nasal-type,” the latest (5th) edition has dropped the qualifier “nasal-type” to acknowledge that the disease can also manifest in other locations, such as the lungs, gastrointestinal tract, and skin [1-3]. Due to rapid local progression and distant metastasis, NKTL is typically characterized by a poor prognosis [4], with the extranasal type being particularly aggressive [5]. The clinical management of this disease remains highly challenging. National Comprehensive Cancer Network guidelines recommend radiotherapy (RT) in the first-line treatment of early-stage extranodal NK/T-cell lymphoma (ENKL). For advanced-stage nasal type and the extranasal type with a poor prognosis, the primary treatment is chemotherapy.

NKTL constitutes approximately 10% of all mature T/NK cell lymphomas globally, with significantly higher prevalence rates in Asia and Central or South America [5,6]. Data from the International Peripheral T-cell Lymphomas database indicate that out of 1153 peripheral T-cell lymphoma cases, ENKL represents 11.8%, with a much higher relative frequency in Asian countries compared to Western nations (22% vs 5%) [5]. Studies have shown that variations in EBV strains (eg, LMP1 30-base-pair deletion) and genetic profiles (eg, STAT3 mutations) may differentially influence the pathogenesis mechanisms across different ethnic groups [7,8]. Most published literature on NKTL consists of case reports, with larger cohort studies mainly originating from China and other Asian regions [1-3,5,9,10]. In contrast, data on NKTL in Western countries remain scarce [11,12]. Using the Surveillance, Epidemiology, and End Results (SEER) database, this study investigates the incidence, demographic and clinical characteristics, treatment options, patient survival, and prognostic factors of NKTL in the United States, with a particular focus on the impact of radiotherapy and chemotherapy on survival, aiming to provide a comprehensive overview of NKTL management and outcomes in this region.

## Methods

### Data Source, Patient Inclusion Criteria, and Study Objectives

This study conducted a retrospective analysis using cohort data from the National Cancer Institute’s SEER 17 registries [13]. The patient cohort was accessed via the SEER website [13] using the SEER*Stat software (version 8.4.3; National Cancer Institute). The selected dataset was “SEER Database: Incidence - SEER Research Data, 17 Registries, Nov 2022 Sub (2000‐2020),” which was released in April 2023, based on submissions from November 2022. The dataset encompasses population-based patients with cancer from various US regions including San Francisco-Oakland Standard Metropolitan Statistical Area, Connecticut, Hawaii, Iowa, New Mexico, Seattle (Puget Sound), Utah, Atlanta (Metropolitan), San Jose-Monterey, Los Angeles, Alaska Natives, Rural Georgia, California excluding San Francisco-Oakland Standard Metropolitan Statistical Area/San Jose-Monterey/Los Angeles, Kentucky, Louisiana, New Jersey, and Greater Georgia, covering approximately 26.5% of the US population (based on the 2020 census).

Patient inclusion criteria were as follows: (1) diagnosis of NK/T-cell lymphoma, nasal-type, classified under the International Classification of Diseases for Oncology, Third Edition (ICD-O-3) code 9719/3; (2) diagnosis made between the years 2000 and 2020; and (3) diagnosis confirmed by positive histopathology. Patients were excluded if they had missing survival information, nonhistological diagnostic confirmation, or were diagnosed solely by autopsy or death certificate.

The objective of this study was to analyze the incidence rate (IR), demographic and clinical characteristics, treatment modalities, survival outcomes, and factors influencing the prognosis of patients with NKTL across 17 US regions from 2000 to 2020. This comprehensive overview aims to enhance our understanding of the diagnosis, treatment, and survival of this rare disease in the United States, particularly evaluating the impact of radiotherapy and chemotherapy on the survival of patients with NKTL.

### Patient Cohort Variables

The variables collected for each patient in this study included the year of diagnosis, gender, race, age at diagnosis (up to ≥90 y), marital status, median household income, and area of residence. Patients were stratified by the year of diagnosis into 4 groups (2000‐2005, and subsequent 5-year intervals), by age into 3 groups (≤39 y, 40‐69 y, and ≥70 y), and by median household income at diagnosis into 3 groups (≥US $70,000, US $50,000‐69,999, and <US $50,000). Racial categories comprised non-Hispanic White (NHW), non-Hispanic Black (NHB), Hispanic, non-Hispanic Asian or Pacific Islander (NHAPI), and non-Hispanic American Indian/Alaska Native (NHAIAN). Marital status was categorized as married or unmarried, with unmarried encompassing separated, widowed, divorced, single, unmarried, or domestic partner. The area of residence was divided into counties in metropolitan areas and nonmetropolitan counties.

Patient disease characteristics included the site of tumor involvement, clinical stage, presence of systemic symptoms (referred to as B symptoms), whether NKTL was the first tumor, as well as treatments received, such as radiotherapy and chemotherapy, and survival outcomes. Sites of tumor involvement were classified as nodal or extranodal, with further details on specific sites. Staging was based on the Ann Arbor Stage and Combined Summary Stage, grouping patients into Ann Arbor stage I (localized), stage II (regional), and stages III or IV (distant). Systemic symptoms were identified through the presence of fever, night sweats, or weight loss, classified under the variable B symptoms recode (B symptoms). Treatment modalities included radiotherapy, categorized by type as beam radiation, radiation NOS (ie, method or source not otherwise specified), and radioactive implants (including brachytherapy), with patients classified as receiving radiotherapy (yes) or not (no/unknown). Chemotherapy was recorded as yes or no/unknown. Survival data encompassed “vital status” and “survival months.” In cases where NKTL was the primary tumor, lymphoma-specific deaths were analyzed using the “SEER cause-specific death classification.”

### Ethical Considerations

The SEER database is a public cancer database, which is authorized by the National Cancer Institute, National Institutes of Health, US Department of Health and Human Services, and USA.gov. The data in the SEER database is deidentified and publicly available, containing no personal identifying information. According to the exemption criteria outlined in 45 CFR 46.104(d)(4) published by the US Department of Health and Human Services, the use of deidentified public data does not require institutional review board approval. In our study, we have been granted permission to use the SEER data and this analysis was exempt from institutional review board review.

### Statistical Analysis

Incidence rates (IR; cases per 100,000) were calculated using data from 2000 to 2020 obtained from the SEER database and were age-adjusted to the US standard population. These calculations were performed using the SEER*Stat 8.4.3 software, which provided the rate and the corresponding 95% CI. Counts and percentages were used for categorical variables, while medians and interquartile ranges (IQR) were used for continuous variables to illustrate baseline patient characteristics. The Pearson *χ*^2^ test and the Wilcoxon rank-sum test were used for comparing categorical and continuous variables between groups, respectively. Overall survival (OS) was analyzed up to 2020 using the Kaplan-Meier estimator, and survival outcomes were compared using the log-rank test. Both univariate and multivariate Cox proportional hazards regression models were applied to identify predictors that may influence OS, presenting hazard ratios (HRs) and 95% CIs. For patients with NKTL as the initial tumor, lymphoma-specific OS was also analyzed. Deaths attributable to NKTL were treated as events, while all other deaths or survivals were considered censored. A *P* value of less than .05 was considered statistically significant. Statistical analyses and graphical representations were conducted using IBM SPSS (version 26.0; IBM Corp) and the R programming language (version 4.4.0; R Foundation for Statistical Computing).

## Results

### Incidence Rate

**Figure 1.**
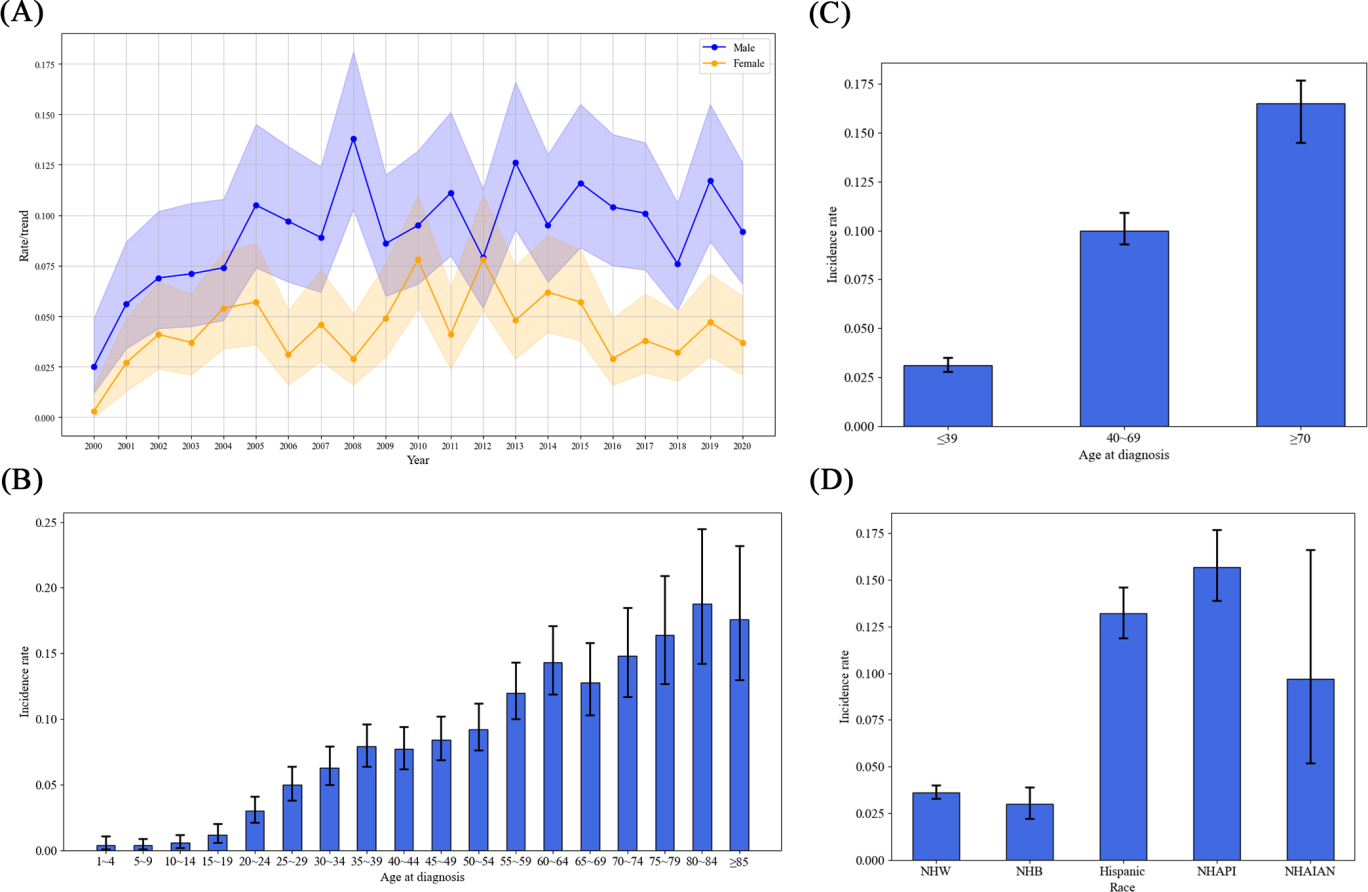
Annual incidence of NKTL in 17 regions of the United States between 2000 and 2020. (**A**) Annual incidence rates for males and females, with lighter shading showing 95% CI. (**B**) Incidence rates for populations of different ages. (**C**) Incidence rates for populations in age groups ≤39 years, 40-69 years, and ≥70 years. (**D**) Incidence rates for different races. NHAIAN: non-Hispanic American Indian/Alaska Native; NHAPI: non-Hispanic Asian or Pacific Islander; NHB: non-Hispanic Black; NHW: non-Hispanic White; NKTL: natural killer/T-cell lymphoma.

From 2000 to 2020, the mean annual IR of NKTL was 0.067 per 100,000 (95% CI 0.063‐0.071). The lowest IR occurred in 2000, at 0.014 per 100,000 (95% CI 0.007‐0.025), and the highest in 2010, at 0.086 per 100,000 (95% CI 0.067‐0.109). The IR among males was significantly higher at 0.093 per 100,000 (95% CI 0.086‐0.100) compared to females at 0.044 per 100,000 (95% CI 0.040‐0.049; *P*<.05), resulting in a male-to-female ratio of 2.11:1. Figure 1A displays the annual IRs for males and females from 2000 to 2020. IRs also showed a gradual increase with age, as depicted in Figure 1B. There were significant differences in IRs across the age groups: ≤39 years at 0.031 per 100,000 (95% CI 0.028‐0.035), 40‐69 years at 0.100 per 100,000 (95% CI 0.093‐0.109), and ≥70 years at 0.165 per 100,000 (95% CI 0.145‐0.187*; P*<.05) (Figure 1C). When analyzing IRs by race, NHAPI had the highest rate at 0.157 per 100,000 (95% CI 0.139‐0.177), similar to Hispanic people at 0.132 per 100,000 (95% CI 0.119‐0.146) and NHAIAN at 0.097 per 100,000 (95% CI 0.052‐0.166). However, these rates were significantly higher than those of NHW and NHB, which were 0.036 per 100,000 (95% CI 0.033‐0.040) and 0.030 per 100,000 (95% CI 0.022‐0.039), respectively, with no significant difference noted between NHW and NHB (*P*<.05) (Figure 1D).

### Demographic and Clinical Characteristics of Patients With NKTL

The study encompassed 1162 participants. Details regarding the demographic and clinical characteristics of the participants are presented in Table 1. Of these, 764 patients (65.7%) were male, resulting in a male-to-female ratio of 1.92, and the median age was 54 (IQR 49‐72) years. The racial demographic was predominated by Hispanic participants (36.5%), followed by NHW (34.6%) and NHAPI (22.9%). The median ages varied significantly across racial groups (*P*<.001): 60.5 years for NHW (IQR 49‐72 y), 51.0 years for NHB (IQR 39‐60 y), 43.0 years for Hispanic people (IQR 31‐56.8 y), 59.0 years for NHAPI (IQR 46.8‐71.0 y), and 44.0 years for other races (IQR 25.5‐57.5 y). A majority of the patients (91.1%) resided in metropolitan areas, 52.7% reported household incomes above US $70,000, and 57.6% were married.

In our cohort, a significant majority of the patients (1016 cases, 87.4%) experienced primary invasion of extranodal tissues. The most frequently affected extranodal site was the nasal cavity (581 cases, 50%), followed by the oropharynx, nasopharynx, pharynx, and throat (164 cases, 14.1%), with the paranasal sinus being the third most common site (88 cases, 7.6%). Patients with invasions in these 3 primary locations, totaling 833 cases (71.7%), were classified as having the nasal type. Other cases involving the gastrointestinal tract, lungs, bone marrow, and skin soft tissues were categorized as extranasal type. Based on the Ann Arbor staging system, when combined with the summary stage, patients were classified as follows: stage I (localized) comprised 490 patients (37.8%), stage II (regional) included 231 patients (17.8%), and stages III and IV (distant) accounted for 489 patients (37.7%). Patients diagnosed after 2010 had records of B symptoms, with 201 (17.4%) exhibiting these symptoms. Regarding treatment, 829 patients (71.3%) underwent chemotherapy, 615 (42.3%) received radiotherapy, and 477 (41%) were treated with a combination of radiotherapy and chemotherapy.

**Table 1.** Demographic and clinical characteristics of patients with NKTL (United States; 2000‐2020; N=1162).

Variable	Number of participants (%)
**Year of diagnosis**	
2000-2005	226 (19.4)
2006-2010	294 (25.3)
2011-2015	338 (29.1)
2016-2020	304 (26.2)
**Gender**	
Female	398 (34.3)
Male	764 (65.7)
**Age group (years)**	
≤39	294 (25.3)
40-69	624 (53.7)
≥70	244 (21)
**Race**	
NHW^a^	402 (34.6)
NHB^b^	53 (4.6)
Hispanic	424 (36.5)
NHAPI^c^	266 (22.9)
NHAIAN^d^	14 (1.2)
Unknown	3 (0.3)
**Marital status at diagnosis**	
Married	669 (57.6)
Unmarried^e^	429 (36.9)
Unknown	64 (5.5)
**Median household income (US $)**	
>70,000	612 (52.7)
50,000-69,999	483 (41.6)
<50,000	67 (5.8)
**Rural-urban continuum**	
Counties in metropolitan areas^f^	1067 (91.1)
Nonmetropolitan counties^g^	91 (7.9)
Unknown	4 (0.3)
**Sites of tumor involvement**	
***Nodal or extranodal***	
Nodal	146 (12.6)
Extranodal	1016 (87.4)
***Specific sites***
Nasal cavity	581 (50)
Paranasal sinus	88 (7.6)
Oropharynx, nasopharynx, pharynx, and throat	164 (14.1)
Gastrointestinal tract	44 (3.8)
Respiratory tract, pleura, and mediastinum	18 (1.5)
Bone, bone marrow	10 (0.9)
Lymph nodes, spleen, and Waldeyer’s ring	146 (12.6)
Skin, soft tissue	62 (5.3)
Others^h^	36 (3.1)
Unknown^i^	13 (1.1)
**Stage**	
Stage I/localized	492 (42.3)
Stage II/regional	231 (19.9)
Stage III or IV/distant	373 (32.1)
Unknown	66 (5.7)
**B symptoms**	
No	383 (33)
Yes	201 (17.3)
Unknown	578 (49.7)
**First malignant or not**	
Yes	1049 (90.3)
No	113 (9.7)
**Radiation**	
Yes^j^	615 (42.3)
No/unknown^k^	547 (47.1)
**Chemotherapy**	
Yes	829 (71.3)
No/unknown	333 (28.7)
**Survival status**	
Survival	460 (39.6)
Death	702 (60.4)

a NHW: non-Hispanic White.

b NHB: non-Hispanic Black.

c NHAPI: non-Hispanic Asian or Pacific Islander.

d NHAIAN: non-Hispanic American Indian/Alaska Native.

e Single (never married), widowed, divorced, separated, unmarried, or domestic partner.

f Counties in metropolitan areas of 1 million population, counties in metropolitan areas of 250,000 to 1 million population, and counties in metropolitan areas of 250,000 population.

g Nonmetropolitan counties adjacent to a metropolitan area, and nonmetropolitan counties not adjacent to a metropolitan area.

h Mammary gland, male/female reproductive system, kidney, lacrimal gland, orbit, central nervous system, thyroid gland, adrenal gland.

i Head, face or neck, not otherwise specified and unknown.

j Beam radiation, radiation, not otherwise specified method, source not specified, or radioactive implants.

k Refused (these data have been recorded by SEER since 1988), recommended, unknown if administered, and none/unknown.

### Survival Analysis

**Figure 2.**
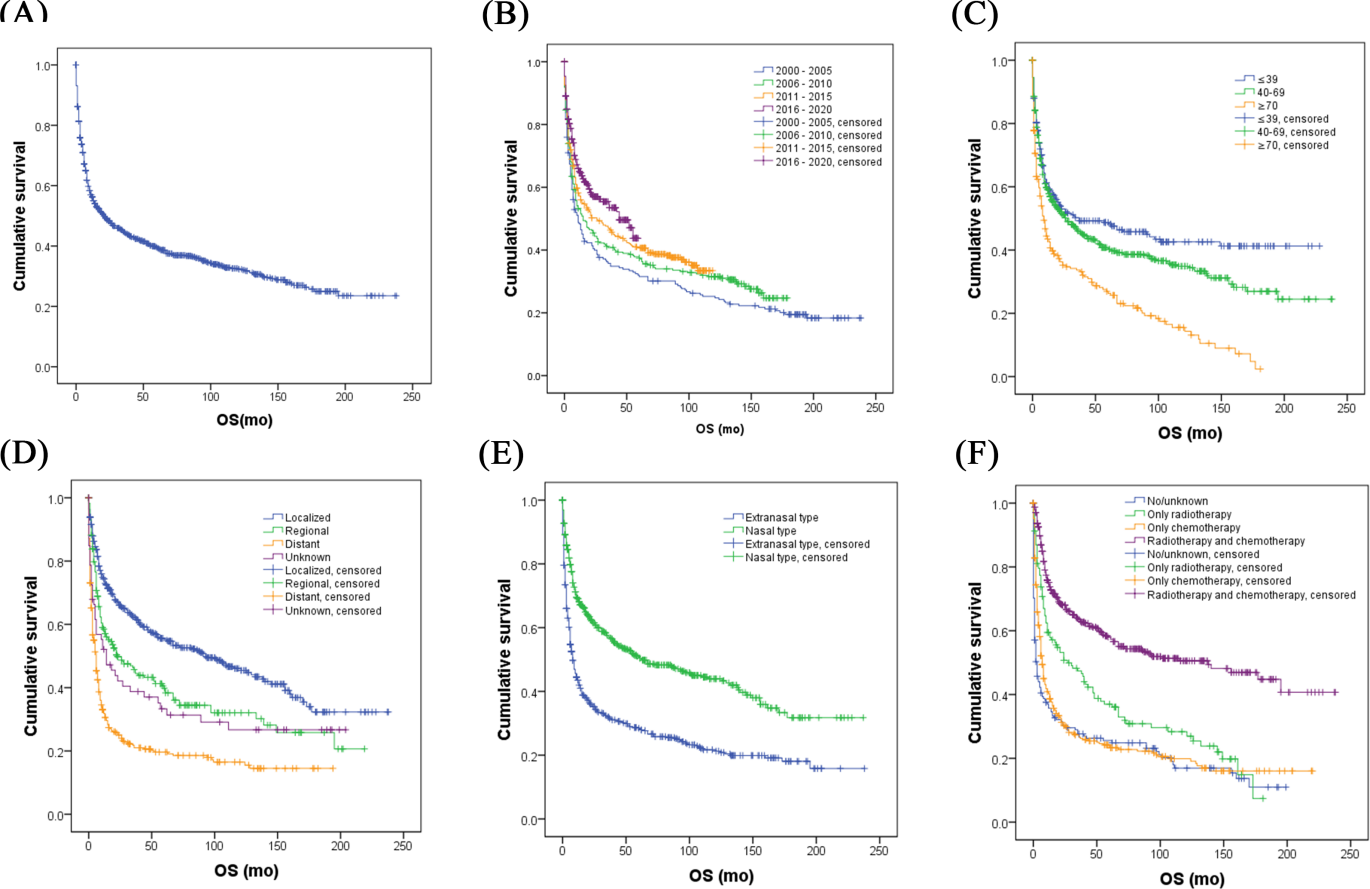
OS for different subgroups of patients with NKTL in 17 regions of the United States between 2000 and 2020. (**A**) OS of 1162 patients with NKTL. (**B**) OS of patients based on different diagnostic years. (**C**) OS of patients with different age groups. (**D**) OS of patients based on different clinical stages. (**E**) OS of patients with nasal type and extranasal type. (**F**) OS of patients based on different treatment modalities. NKTL: natural killer/T-cell lymphoma; OS: overall survival.

By the end of the follow-up period in December 2020, the median follow-up duration was 12.5 months (IQR 3.0‐59.0 mo), with a total of 702 deaths recorded, representing 60.4% of the cohort. The median survival time was 21.0 months (95% CI 14.5‐27.5). The OS rates were 56.3% at 12 months, 44.5% at 35 months, and 39.5% at 60 months, as shown in Figure 2A.

Survival analyses for different subgroups of patients are detailed in Table 2 and Table S1 in Multimedia Appendix 1. When grouped by year of diagnosis, there was a significant improvement in survival over the years (*P*<.001). The 5-year OS rates for patients diagnosed in the periods 2000‐2005, 2006‐2010, and 2011‐2015 were 31.5%, 37.5%, and 41.0%, respectively (Figure 2B). Age also played a crucial role in influencing survival outcomes. Compared to patients aged ≤39 years and those aged 40‐69 years, older patients (≥70 y) exhibited markedly poorer survival, with a 5-year OS of only 26.3% (*P*<.001 for both comparisons); the 5-year OS rates for the younger age groups were 48.7% and 40.5%, respectively; there was no significant difference observed between them (*P*=.07), as illustrated in Figure 2C. No significant differences in survival were observed based on gender or race (*P*=.61 and *P*=.44, respectively). Additionally, factors such as marital status and area of residence at the time of diagnosis did not significantly impact survival outcomes. However, household income at diagnosis appeared to influence survival to some extent (*P*=.04).

Survival was significantly better in patients diagnosed with NKTL as their initial tumor compared to those who had other types of tumors previously. The median OS for patients with NKTL as their first tumor was 26.0 months (95% CI 18.1‐33.9 mo), whereas it was only 11.0 months for patients with prior tumors (95% CI 6.1‐15.9 mo*; P*<.001). Further analysis revealed a notable age difference between the two groups: the median age for patients with NKTL as their first tumor was 52 years, compared to 71 years for those with nonfirst NKTL, indicating a significant disparity (*P*<.001). This variation in survival rates may be influenced by the patients’ ages.

Patients with localized disease exhibited significantly better OS than those with regional or distant disease (*P*<.001). There was also a marked survival difference between localized, regional, and distant stages (*P*<.001), with 5-year OS rates of 55.3%, 39.8%, and 19.6%, respectively (Figure 2D). Furthermore, patients with primary extranodal invasion had significantly better survival compared to those with nodal invasion (*P*<.001), featuring a median OS of 27.0 months (95% CI 17.9‐36.1 mo) versus 6.0 months (95% CI 4.1‐7.9 mo). Patients diagnosed with the nasal type of the disease showed significantly better survival outcomes than those with the extranasal type, as detailed in Table 2, Table 1S in Multimedia Appendix 1, and Figure 2E. The 3-year and 5-year OS rates for nasal type were 52.5% and 46.4%, respectively, whereas for extranasal type, they were substantially lower at 24.4% and 22.2%, respectively.

**Table 2.** Survival analysis for different subgroups of patients with natural killer/T-cell lymphoma (United States; 2000‐2020; N=1162).

Variable	Median overall survival	1-year overall survival rate, %	3-year overall survival rate, %	*P* value
**Year of diagnosis**				<.001
2000-2005	11.0 (6.7‐15.3)	48.3	35.8	
2006-2010	15.0 (7.4‐22.6)	52.9	40.8	
2011-2015	26.0 (12.4‐39.7)	57.2	46.7	
2016-2020	44.0 (28.4‐59.6)	65.1	53.5	
**Gender**				.61
Female	20.0 (8.8‐31.2)	56.7	45.6	
Male	22.0 (14.6‐29.4)	56.1	43.9	
**Age (years)**				<.001
≤39	37.0 (0‐79.8)	60.9	50.2	
40-69	27.0 (17.5‐36.5)	59.1	46	
≥70	9.0 (6.5‐11.5)	43.5	33.7	
**Race (n=1159)**				.44
NHW^a^	18.0 (8.3‐27.7)	54.2	43.9	
NHB^b^	22.0 (0‐45.5)	56.1	40	
Hispanic	23.0 (11.4‐34.6)	58.4	45.5	
NHAPI^c^	22.0 (7.9‐36.1)	57.4	45.5	
NHAIAN^d^	4.0 (0‐13.2)	35.7	28.6	
**Marital status at diagnosis (n=1098)**				.98
Married	24.0 (14.8‐33.2)	56.7	45.8	
Unmarried^e^	18.0 (9.6‐26.4)	55.5	43.4	
Unknown	21.0 (5.8‐36.2)	56.4	42	
**Median household income (US $)**				.04
>70,000	27.0 (15.6‐38.4)	59.3	46.5	
50,000-69,999	18.0 (10.9‐25.1)	53.6	42.5	
<50,000	12.0 (3.1‐20.9)	48.8	41	
**Rural-urban continuum (n=1158)**				.16
Counties in metropolitan areas^f^	22.0 (15.5‐28.5)	56.9	44.7	
Nonmetropolitan counties^g^	13.0 (0‐29.4)	50.7	42.6	
**First malignant or not**				<.001
Yes	26.0 (18.1‐33.9)	57.2	46.1	
No	11.0 (6.1‐15.9)	48	30.1	
**Clinical stage**				<.001
Stage I/localized	93.0 (61.2‐124.8)	74.4	62.4	
Stage II/regional	23.0 (9.6‐36.4)	58.5	45.7	
Stage III or IV/distant	6.0 (4.8‐7.2)	31.8	21	
Unknown	14.0 (2.2‐25.8)	52.1	38.8	
**Nodal or extranodal**				<.001
Nodal	6.0 (4.1‐7.9)	35	23.3	
Extranodal	27.0 (17.9‐36.1)	59.3	47.5	
**Nasal type (n=1149)**				<.001
No	6.0 (4.5‐7.5)	36.5	24.4	
Yes	44.0 (30.8‐57.2)	64	52.5	
**B symptoms**				<.001
No	67.0 (40.0‐94.0)	69.4	57.6	
Yes	11.0 (3.4‐18.6)	49.1	41	
Unknown	13.0 (9.5‐16.5)	50.2	37.6	
**Radiotherapy**				<.001
Yes^h^	67.0 (38.3‐95.7)	71.6	59.8	
No/unknown^i^	6.0 (4.7‐7.3)	38.6	26.9	
**Chemotherapy**				<.001
Yes	30.0 (18.2‐41.8)	60.5	47.9	
No/unknown	9.0 (5.2‐12.8)	45.7	36	
**Treatment**				<.001
No/unknown	3.0 (1.4‐4.6)	35.8	27.7	
Only radiation	27.0 (10.7‐43.3)	59.4	47.4	
Only chemotherapy	7.0 (5.6‐8.4)	40.2	26.6	
Radiation and chemotherapy	138.0 (76.0‐200.0)	75.2	63.5	

a NHW: non-Hispanic White.

b NHB: non-Hispanic Black.

c NHAPI: non-Hispanic Asian or Pacific Islander.

d NHAIAN: non-Hispanic American Indian/Alaska Native.

e Single (never married), widowed, divorced, separated, unmarried, or domestic partner.

f Counties in metropolitan areas of 1 million population, counties in metropolitan areas of 250,000 to 1 million population, and counties in metropolitan areas of 250,000 population.

g Nonmetropolitan counties adjacent to a metropolitan area, and nonmetropolitan counties not adjacent to a metropolitan area.

h Beam radiation, radiation, not otherwise specified method, source not specified, or radioactive implants.

i Refused (these data have been recorded by SEER since 1988), recommended, unknown if administered, and none/unknown.

Among the localized patients, those with the nasal type (420 patients, 85.5%) had a notably higher median OS of 121.0 months (95% CI 89.1‐152.9 mo), with 3-year and 5-year OS rates of 67.4% and 59.7%, respectively. In contrast, the 71 patients (14.5%) with the extranasal type experienced a significantly shorter median OS of only 13.0 months (95% CI 6.6‐19.3 mo), with 3-year and 5-year OS rates of 31.7% and 28.0%, respectively. In stages III and IV (distant), patients with the nasal type also showed superior survival outcomes compared to those with the extranasal type. The median OS for the nasal type was 7.0 months (95% CI 5.2‐8.8 mo), significantly better than the median OS for the extranasal type, which was 5.0 months (95% CI 3.6‐6.4 mo; *P*=.04). The 3-year and 5-year OS rates for the nasal type were 24.9% and 24.0%, respectively, while those for the extranasal type were lower at 17.5% and 15.6%, respectively.

Further analysis of the survival of patients with specific extranodal invasion sites revealed that those with nasal cavity involvement exhibited the best median OS of 63.0 months (95% CI 33.8‐92.2 mo), significantly outperforming patients with other types of extranodal involvement (*P*<.05). This group was followed by patients with involvement of the oropharynx, nasopharynx, pharynx, and throat, who had a median OS of 14.0 months (95% CI 6.9‐21.1 mo). Patients with paranasal sinus involvement had the third highest median OS at 12.0 months (95% CI 0‐38.1 mo), as shown in Table S2 and Figure S1 in Multimedia Appendix 1. Patients with nodal invasion, including involvement of splenic and Weil’s rings, also experienced poor outcomes, with a median OS of only 6.0 months and a 3-year OS rate of 23.3%. The median OS was even lower in patients presenting with B symptoms at 11.0 months (95% CI 3.4‐18.6 mo), compared to those without B symptoms, who had a median OS of 67.0 months (95% CI 40.0‐94.0 m, *P*<.001).

**Figure 3.**
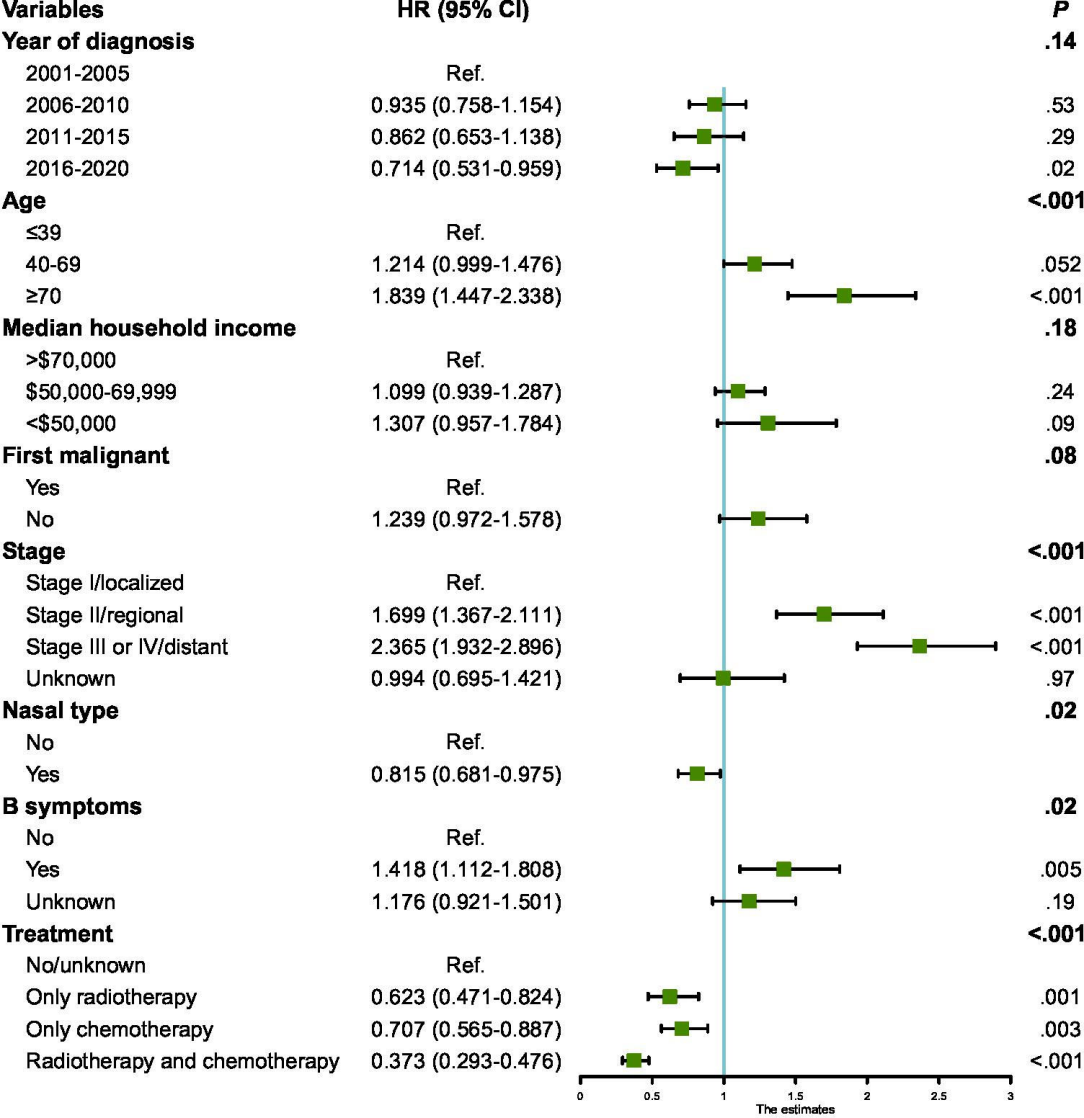
Multivariate Cox proportional hazards regression model to analyze the factors influencing the prognosis of patients with natural killer/T-cell lymphoma. HR: hazard ratio.

Radiotherapy and chemotherapy are primary treatments for NKTL. The analysis indicated that patients who underwent either radiotherapy or chemotherapy exhibited improved survival (*P*<.001). Specifically, 477 patients received both treatments concurrently and demonstrated the highest OS, with a median OS of 138.0 months (95% CI 76.0‐200.0 mo). This outcome was significantly superior to those who underwent only radiotherapy (27.0 mo, 95% CI 10.7‐43.3 mo) or chemotherapy (7.0 mo, 95% CI 5.6‐8.4 mo), and to patients categorized as no/unknown (3.0 mo, 95% CI 1.4‐4.6 mo*; P*<.001) (Table 2, Table S1 in Multimedia Appendix 1, Figure 2F). Five-year OS rates for these groups were 58.0%, 37.0%, 23.3%, and 25.6%, respectively. Additionally, survival for patients treated with only radiotherapy was also significantly higher than for those who received only chemotherapy or no treatment (*P*=.001 and *P*<.001, respectively). Survival rates for patients undergoing chemotherapy alone were also significantly higher than for those receiving no treatment (*P*=.02). Multivariate Cox regression analysis incorporated variables such as year of diagnosis, age at diagnosis, annual household income, whether it was the first tumor, clinical stage, nasal type, presence of B symptoms, and treatment types. Results identified several independent prognostic factors for NKTL. Age at diagnosis ≥70 years (HR=1.839; *P*<.001), advanced clinical stages II (HR=1.699; *P*<.001) and III/IV (HR=2.365; *P*<.001), nasal type (HR=0.815; *P*=.02), presence of B symptoms (HR=1.418; *P*=.005), radiotherapy (HR=0.623; *P*=.001), chemotherapy (HR=0.707; *P*=.003), and combined radiotherapy and chemotherapy (HR=0.373; *P*<.001) were determined to be significant predictors of survival (Table S1 in Multimedia Appendix 1, Figure 3).

### Clinical Characteristics and Subgroup Survival Analysis of Patients Receiving Radiotherapy and Chemotherapy

Patients receiving both radiotherapy and chemotherapy demonstrated the highest survival rates, with an HR of 0.293 (95% CI 0.237‐0.364). Treatment with radiotherapy or chemotherapy alone also significantly improved survival, with HRs of 0.563 (95% CI 0.435‐0.728) for radiotherapy alone and 0.808 (95% CI 0.659‐0.989) for chemotherapy alone. Further analysis of clinical characteristics revealed significant variations based on the type of treatment received. Differences were observed in the year of diagnosis, age at diagnosis, marital status, whether the involvement was extranodal or nodal, nasal type, clinical stage, presence of B symptoms, and whether it was the first tumor (Table 3).

**Figure 4.**
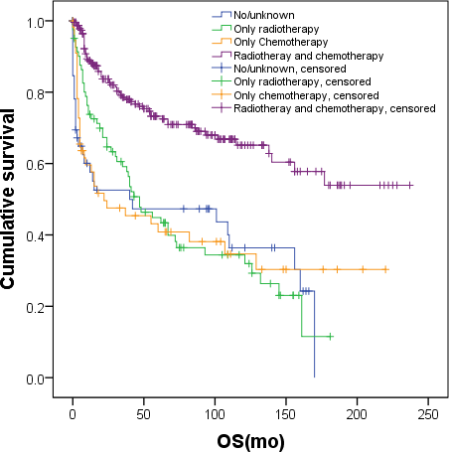
OS of patients with different treatment modalities (combination of radiotherapy and chemotherapy, radiotherapy alone, chemotherapy alone, or neither treatment). OS: overall survival.

We created subgroups of patients based on factors that might influence the clinical treatment decisions of hematologists treating malignancies, examining the impact of receiving radiotherapy, chemotherapy, or a combination of both on survival across these subgroups. These subgroups were defined by patients’ age, clinical stage, and nasal type status. The results are detailed in Table 4. Patients with varying clinical stages, different age groups, and whether they were of the nasal type or not, who received a combination of radiotherapy and chemotherapy, exhibited the best survival (*P*<.001). Notably, among stage I/localized patients with nasal type—a total of 420 patients—those receiving combination therapy (231 patients, 55%), radiotherapy alone (81 patients, 19.3%), chemotherapy alone (56 patients, 13.3%), and neither therapy (52 patients, 12.4%) had median OS rates of not reached, 47.0 months (95% CI 30.2‐63.7 mo), 22.0 months (95% CI 0‐61.1 mo), and 40.0 months (95% CI 0‐153.2 mo), respectively. These differences were statistically significant (*P*<.001). Survival was significantly superior in the combination therapy group compared to the other 3 groups. However, there were no significant differences in survival among the latter 3 groups (Figure 4). Five-year OS rates were 73.3% in the combination therapy group, 44.9% in the radiotherapy alone group, and 40.8% in the chemotherapy alone group.

Among the 373 patients with advanced, distant-stage disease, treatment modalities were distributed as follows: 85 patients (22.8%) received combined radiotherapy and chemotherapy, 22 patients (5.9%) received radiotherapy alone, 195 patients (52.3%) underwent chemotherapy alone, and 71 patients (19.0%) received neither treatment. The median overall survival (OS) for these groups was 11.0 months (95% CI 7.0‐15.0 mo), 3.0 months (95% CI 0‐7.5 mo), 6.0 months (95% CI 4.4‐7.6 mo), and 1.0 month (95% CI 0.4‐1.6 mo), respectively, demonstrating significant differences (*P*<.001). The 1-year OS rates were 48.4% for combined therapy, 20.2% for radiotherapy alone, 31.4% for chemotherapy alone, and 16.3% for no treatment. Patients receiving combination therapy exhibited significantly better survival compared to those in the other 3 groups. The 5-year OS for the combination therapy group was 34.7%. There was no significant difference in survival among the other 3 groups (Figure 4).

**Table 3.** Demographic and clinical characteristics of patients with natural killer/T-cell lymphoma based on the clinical treatment decisions (United States; 2000‐2020; N=1162).

Variable	Radiotherapy and chemotherapy, n (%)	Only radiotherapy, n (%)	Only chemotherapy, n (%)	No/unknown, n (%)	*P* value
**Year of diagnosis**					<.001
2000-2005	80 (35.4)	18 (8)	82 (36.3)	46 (20.4)	
2006-2010	97 (33)	54 (18.4)	86 (29.3)	57 (19.4)	
2011-2015	149 (44.1)	45 (13.3)	90 (26.6)	54 (16)	
2016-2020	151 (49.7)	21 (6.9)	94 (30.9)	38 (12.5)	
**Gender**					.28
Female	161 (40.5)	51 (12.8)	110 (27.6)	76 (19.1)	
Male	316 (41.4)	87 (11.4)	242 (31.7)	119 (15.6)	
**Age group (years)**					<.001
≤39	144 (49)	21 (7.1)	90 (30.6)	39 (13.3)	
40-69	277 (44.4)	50 (8)	222 (35.6)	75 (12)	
≥70	56 (23)	67 (27.5)	40 (16.4)	81 (33.2)	
**Race (n=1159)**					.08
NHW^a^	169 (42)	49 (12.2)	111 (27.6)	73 (18.2)	
NHB^b^	14 (16.4)	4 (7.5)	25 (47.2)	10 (18.9)	
Hispanic	186 (43.9)	49 (11.6)	133 (31.4)	56 (13.2)	
NHAPI^c^	101 (38)	35 (13.2)	80 (30.1)	50 (18.8)	
NHAIAN^d^	7 (50)	1 (7.1)	2 (14.3)	4 (28.6)	
**Marital status at diagnosis (n=1098)**					<.001
Married	300 (44.8)	83 (12.4)	205 (30.6)	81 (12.1)	
Unmarried^e^	163 (38)	48 (11.2)	136 (31.7)	82 (19.1)	
**Median household income (US $)**					.43
>70,000	266 (43.5)	68 (11.1)	188 (30.7)	90 (14.7)	
50,000-69,999	185 (38.3)	62 (12.8)	144 (29.8)	92 (19)	
<50,000	26 (38.8)	8 (11.9)	20 (29.9)	13 (19.4)	
**Rural-urban continuum (n=1158)**					.79
Counties in metropolitan areas^f^	437 (41)	127 (11.9)	327 (30.6)	176 (16.5)	
Nonmetropolitan counties^g^	38 (41.8)	11 (12.1)	24 (26.4)	18 (19.8)	
**Nodal or extranodal**					<.001
Nodal	32 (21.9)	3 (2.1)	76 (52.1)	35 (24)	
Extranodal	445 (43.8)	135 (13.3)	276 (27.2)	160 (15.7)	
**Nasal type (n=1149)**					<.001
No (n=316)	59 (18.7)	14 (4.4)	158 (50)	85 (26.9)	
Yes (n=833)	416 (49.9)	124 (14.9)	190 (22.8)	103 (12.4)	
**Stage (n=1096)**					<.001
Stage I/localized	250 (50.8)	90 (18.3)	85 (17.3)	67 (13.6)	
Stage II/regional	134 (59)	23 (10)	56 (24.2)	18 (7.8)	
Stage III or IV/distant	85 (22.8)	22 (5.9)	195 (52.3)	71 (19)	
**B symptoms**					<.001
No	213 (55.6)	48 (12.5)	81 (21.1)	41 (10.7)	
Yes	79 (39.3)	14 (7)	81 (40.3)	27 (13.4)	
Unknown	185 (32)	76 (13.1)	190 (32.9)	127 (22)	
**First malignant or not**					<.001
Yes	444 (42.3)	327 (31.2)	114 (10.9)	164 (15.6)	
No	33 (29.2)	25 (22.1)	24 (21.2)	31 (27.4)	

a NHW: non-Hispanic White.

b NHB: non-Hispanic Black.

c NHAPI: non-Hispanic Asian or Pacific Islander.

d NHAIAN: non-Hispanic American Indian/Alaska Native.

e Single (never married), widowed, divorced, separated, unmarried, or domestic partner.

f Counties in metropolitan areas of 1 million population, counties in metropolitan areas of 250,000 to 1 million population, and counties in metropolitan areas of 250,000 population.

g Nonmetropolitan counties adjacent to a metropolitan area, and nonmetropolitan counties not adjacent to a metropolitan area.

**Table 4.** Survival analysis for different subgroups of patients with natural killer/T-cell lymphoma based on the clinical treatment decisions (United States; 2000‐2020; N=1162).

Variable		Radiotherapy and chemotherapy, median (95% CI)	Only radiotherapy, median (95% CI)	Only chemotherapy, median (95% CI)	No/unknown, median (95% CI)	*P* value
**Clinical stage**						
	Stage I/localized	Not reached	47.0 (37.5‐56.5)	13.0 (5.6‐20.4)	15.0 (0‐48.8)	<.001
	Stage II/regional	52.0 (25.9‐78.1)	9.0 (0.5‐17.5)	10.0 (3.0‐17.0)	1.0 (0‐2.4)	<.001
	Stage III or IV/distant	11.0 (7.0‐15.0)	3.0 (0‐7.5)	6.0 (4.4‐7.6)	1.0 (0.4‐1.6)	<.001
	Unknown	57.0 (0‐162.8)	6.0 (0‐14.0)	21.0 (0‐42.1)	6.0 (0‐22.5)	.22
**Age group (years)**						
	≤39	Not reached	93.0 (0‐199.6)	8.0 (3.1‐12.9)	7.0 (0‐33.8)	<.001
	40-69	138.0 (77.2‐198.8)	18.0 (0‐37.5)	8.0 (6.3‐9.7)	4.0 (0‐9.2)	<.001
	≥70	59.0 (20.0‐98.0)	38.0 (12.9‐63.1)	6.0 (3.7‐8.3)	1.0 (0.2‐1.8)	<.001
**Site of involvement**						
	Nasal type	156.0 (100.6‐211.4)	35.0 (17.5‐52.5)	9.0 (4.7‐13.3)	7.0 (0‐15.3)	<.001
	Extranasal type	15.0 (0.1‐29.9)	7.0 (1.5‐12.5)	6.0 (4.2‐7.8)	1.0 (0‐2.0)	<.001

### Lymphoma-Specific Survival

**Figure 5.**
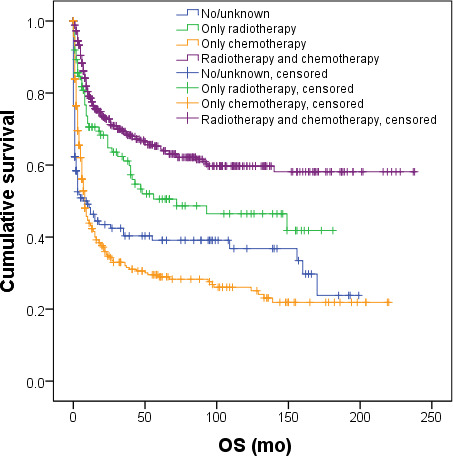
Lymphoma-specific survival in patients with different treatment modalities (combination of radiotherapy and chemotherapy, radiotherapy alone, chemotherapy alone, or neither treatment). OS: overall survival.

NKTL was the initial diagnosis for 1049 patients, of whom 17 had unknown causes of death. Consequently, 1032 patients were included in the lymphoma-specific mortality analysis. By the end of the follow-up period, 491 (47.6%) of these patients had succumbed to NKTL, with a median lymphoma-specific OS of 50.0 months (95% CI 27.3‐72.7 mo). An analysis of patient survival based on their treatment modalities—radiotherapy and chemotherapy—revealed that those who received combination therapy had superior survival compared to those treated with radiotherapy alone, chemotherapy alone, or neither treatment (*P*=.011, *P*<.001, and *P*<.001, respectively). The median OS for these groups was not reached, 72.0 months (95% CI 0‐153.5 mo), 8.0 months (95% CI 6.0‐10.0 mo), and 10.0 months (95% CI 2.1‐17.8 mo), respectively. Furthermore, patients who underwent radiotherapy alone experienced significantly better survival compared to those who underwent chemotherapy alone and those who did not receive either treatment (*P*<.001 for both comparisons). However, there was no significant difference in survival between patients who received chemotherapy alone and those who did not receive any treatment (*P*=.73) (Figure 5).

## Discussion

### Principal Findings

In this study, we observed that the incidence rate of NKTL increased with age, and there was no significant change in the incidence of the disease between 2000 and 2020, although notable differences in incidence among different racial groups were identified. The use of combined radiotherapy and chemotherapy markedly improved survival rates, even in patients with limited nasal type or advanced disease. To the best of our knowledge, this study is the first to successfully present a comprehensive overview of NKTL management and outcomes in the United States.

NKTL is a rare lymphoma originating from mature T/NK cells. According to data from the SEER 17 database, the average annual incidence rate across 17 regions in the United States was 0.067 per 100,000 between 2000 and 2020, with the rate remaining relatively stable over the past 2 decades. The incidence rate was significantly higher in males than in females, with a ratio of approximately 2.11:1. Additionally, the incidence rate gradually increased with age, without showing peaks in any specific age groups. Racial disparities in incidence rates were noted; NHAPI and Hispanic populations had higher rates ranging from 0.132 to 0.157 per 100,000, whereas NHW and NHB populations had lower rates around 0.03 per 100,000, consistent with previous reports [1,2,5,14]. The underlying reasons for these differences are not well understood but are hypothesized to be related to variations in EBV subtypes, genetic susceptibility, or both [7,15-17]. There are 2 main strains or branches of EBV currently identified globally, each with multiple variants. Notably, the strains found in Asia differ significantly from those in other regions [18]. Transcriptome analyses have indicated that certain EBV variants are more likely to cause NKTL or other malignancies [19,20]. A recent collaborative effort involving experts from China, the United States, and Mexico conducted comprehensive genomic analyses and related clinical studies on 209 patients with extranodal NKTL. This collaboration led to the identification of 7 distinct gene clusters associated with varying clinical outcomes, revealing notable genetic similarities between Asian and Hispanic patients that suggest a possible shared pathogenesis and tumor development [8]. Furthermore, polymorphisms in genes related to the host immune-inflammatory responses have been linked to NKTL development [16,21]. However, further research is needed to deepen our understanding of the genetic susceptibility associated with NKTL.

Like many cancers, NKTL predominantly affects adults, with most studies reporting a median age of diagnosis between 44 and 54 years. In our cohort, the median age was 54 years. Notably, we observed significant racial differences in the median age at first diagnosis; Hispanic and NHAIAN patients were generally younger, with a median age of less than 45 years, whereas NHW and NHAPI patients had a median age of about 60 years. Our survival analysis identified that being aged 70 years or older at diagnosis was an independent adverse factor affecting patient survival. Moreover, patients diagnosed in more recent years have demonstrated improved survival outcomes. Specifically, for those diagnosed after 2016, the median survival was 44.0 months and the 3-year OS rate was 53.5%. This improvement is likely due to the introduction and use of more effective treatments for NK/T lymphomas, such as L-asparaginase and immune checkpoint inhibitors [1,3].

The Ann Arbor staging system, commonly used for clinical staging of NKTL, suggests significant prognostic implications. In our study, a majority of patients (723 cases, 62.2%) were classified as stage I/II with relatively limited invasion. However, this staging system primarily assesses the extent of lesion involvement and fails to account for the significant prognostic impact of primary invasion of the upper aerodigestive tract by NKTL [22]. To address this, the extranodal NKTL (Chinese-Asia or CA) staging system, developed byChina and Asia Lymphoma Study Group, incorporates factors such as the site of involvement, regional lymph node involvement, and distant metastasis. This system has been validated in patients treated with asparaginase [23]. Unfortunately, due to data acquisition limitations, we were unable to validate this prognostic staging system in our cohort.

The upper aerodigestive tract—including the nasal cavity, nasopharynx, sinuses, and palate—is the most frequent site of NKTL involvement. Patients with primary invasion of these areas are categorized as having ENKL, nasal type. In contrast, primary extranasal invasion sites such as the skin, soft tissues, gastrointestinal tract, and testes, are associated with the extranasal type. This type presents more adverse prognostic clinical features and poorer OS, which has been confirmed by multiple clinical studies [5,6,8,24]. Aggressive NK-cell leukemia was once considered an aggressive subtype of extranodal NKTL with vascular involvement [25,26]. However, in the *5th edition of the WHO Classification of Haematolymphoid Tumours*, it is now recognized as a distinct entity of peripheral T-cell lymphoma, not otherwise specified [27]. This rare lymphoma frequently involves the skin and central nervous system and is associated with a grave prognosis [28]. Due to its unclear pathological nature, this subtype has been excluded from this analysis. Notably, limited nasal or nasopharyngeal involvement is considered the most favorable prognosis group (stage I) within the CA extranodal NKTL staging system. In our patient cohort, the nasal type comprised 71.7%. Recent data from 14 and 19 medical centers in China, involving more than 1000 patients each, showed that the nasal type represented 87.7% (1393/1589) and 86.1% (1006/1168) of cases [3,23]. This prevalence suggests that the nasal type may be more common in Asian populations.

Survival analysis revealed that the median OS of our patients was 21 months, with 3-year and 5-year OS rates of 44.5% and 39.5%, respectively. Patients diagnosed with the nasal type exhibited significantly better prognoses compared to those with the nonnasal type, achieving 5-year OS rates of 46.4% and 22.2%, respectively. Specifically, among patients with localized disease and those with stage III/IV disease, which indicates extensive involvement at other sites, 5-year OS rates for the nasal type were 59.7% and 24.0%, respectively. This contrasts with 5-year OS rates of only 28% and 15.7% for patients with the nonnasal type. Comparatively, literature indicates that the median OS in the Asian population was 76 months, with a 3-year OS of 59% [2]. For patients with limited disease (stage I/II) of the nasal type, the 5-year OS was approximately 53% [22], whereas those with nasal type localized disease achieved a 5-year OS of up to 76% [24], suggesting better survival rates than those observed in our study group.

The present treatment for NKTL still relies on radiotherapy and chemotherapy. A significant portion (71.3%) of patients in this group underwent chemotherapy, while fewer than half received radiotherapy. Among the 477 patients (41%) who underwent both radiotherapy and chemotherapy, the best survival outcomes were observed, with a median OS of 138 months and a 5-year OS rate of 58%. In contrast, patients who received either radiotherapy or chemotherapy alone had lower median survival times of 27 months and 7 months, respectively. A notable difference in survival was observed among these 3 patient groups. Further analysis of patient survival across different treatments was conducted based on age subgroup, clinical stage, and nasal type. The findings indicated that patients who underwent combined radiotherapy and chemotherapy consistently exhibited the best survival outcomes across all subgroups. Even among patients with localized (stage I) nasal type disease, the combination therapy significantly enhanced survival rates. The 5-year OS rate was 73.3% in the combination therapy group, compared to 44.9% and 40.8% in the radiotherapy and chemotherapy alone groups, respectively. Recent studies have shown that in patients with ENKL, particularly those with early limited disease (Ann Arbor stage I/II confined to the nasal cavity), the primary treatment modality involving radiotherapy (either alone or in combination with chemotherapy) resulted in a 5-year OS rate of 70%. Low-risk patients treated solely with radiotherapy achieved particularly favorable outcomes, with a 5-year OS rate approaching 90%, while the addition of chemotherapy did not yield further improvements in patient survival [23,29-31]. Nevertheless, for early-stage patients at moderate to high risk, combining radiotherapy with chemotherapy led to significant enhancements in both OS and progression-free survival compared to radiotherapy alone, with a 5-year OS and progression-free survival of 73.2% versus 60.9% (*P*<.001) and 63.5% versus 54.2% (*P*<.001), respectively. Hence, it is suggested that chemotherapy could be adjunctive to radiotherapy solely for early-stage patients with NKTL facing a grim prognosis [29-33]. Current National Comprehensive Cancer Network guidelines suggest that stage I/II patients with extranodal nasal-type NKTL first be assessed for their fitness for chemotherapy, followed by treatment with radiotherapy alone or through clinical trials. Radiotherapy combined with chemotherapy is only recommended for patients with advanced-stage nasal disease and all patients with extranasal involvement. In light of the above, incorporating risk-adapted therapy into the guidelines is essential to optimize the benefits of combined chemoradiotherapy for early-stage disease. However, additional confirmation through extensive prospective clinical studies with large sample sizes may be warranted.

In patients presenting with nonnasal type or advanced (stage III/IV) disease, treatment regimens containing L-asparaginase, such as SMILE (dexamethasone, methotrexate, ifosfamide, L-asparaginase, and etoposide) and P-GEMOX (pembrolizumab, gemcitabine, and oxaliplatin), have significantly enhanced outcomes when combined with consolidation radiotherapy. The 5-year OS rates for these regimens ranged from 34% to 50% [10,34]. In this patient cohort, those with distant metastasis exhibited a 5-year OS of 19.6%. However, patients who received both radiotherapy and chemotherapy demonstrated markedly improved survival, with a 5-year OS of 34.7%. The integration of radiotherapy and chemotherapy can be administered either simultaneously or sequentially, using a sandwich approach [32,35]. Nevertheless, patients with advanced NKTL who have extensive disease primarily undergo chemotherapy [10,34,36,37].

Other factors that may affect patient survival include annual household income at the time of diagnosis, with patients from higher-income households generally exhibiting better survival rates. Conversely, patients with prior diagnoses of other tumors or those experiencing systemic symptoms tend to have poorer survival outcomes. The multivariate Cox regression analysis identified several independent prognostic factors for patients with NKTL, including patient age, clinical stage, nasal type, systemic symptoms, and the receipt of both radiotherapy and chemotherapy. EBV viral load is considered to have a significant prognostic impact on survival in NKTL. A meta-analysis of 11 studies found that a high pretreatment EBV viral load was associated with worse survival outcomes (HR=3.45; *P*=.001). Furthermore, patients whose EBV DNA levels returned to normal ranges after treatment demonstrated better survival than those with persistently positive EBV DNA levels [35,38,39]. Consequently, measuring EBV DNA viral load through quantitative polymerase chain reaction is deemed valuable for both diagnostic and prognostic purposes and is recommended by the National Comprehensive Cancer Network as part of the initial workup. Unfortunately, the SEER program does not provide this information.

This retrospective study has several inherent limitations. The SEER program lacks critical data such as patients’ physical status, serum lactate dehydrogenase levels, results from other prognostic scoring systems, EBV viral load, and specific treatment regimens. Despite these constraints, the analysis presented in this paper yielded some valuable insights. However, to substantiate these findings, prospective, multicenter studies are necessary.

### Conclusions

Overall, this study indicated that the incidence of NKTL has remained stable in recent years. Patients with the nasal type or the use of combined radiotherapy and chemotherapy generally experience better survival outcomes. The results presented an overview of the incidence rate, demographic and clinical characteristics, treatment regimens, and outcomes of NKTL in the United States, which may provide data support for the precise diagnosis and treatment of this rare disease. Further validation through prospective multicenter clinical trials can be done to confirm these findings.

## Supplementary material

10.2196/70129 Multimedia Appendix 1 Supplementary Tables S1-S2 and Figures S1-S2.

## Abbreviations

EBV: Epstein-Barr virus
ENKL: extranodal natural killer/T-cell lymphoma
HR: hazard ratio
IR: incidence rate
NHAIAN: non-Hispanic American Indian/Alaska Native
NHAPI: non-Hispanic Asian or Pacific Islanders
NHB: non-Hispanic Black
NHW: non-Hispanic White
NK: natural killer
NKTL: natural killer/T-cell lymphoma
OS: overall survival
P-GEMOX: pembrolizumab, gemcitabine, and oxaliplatin
RT: radiotherapy
SEER: Surveillance, Epidemiology, and End Results
SMILE: dexamethasone, methotrexate, ifosfamide, L-asparaginase, and etoposide
