# Supplementary material for: Incidence, Prognostic Factors, and Treatment Impact on Survival in Natural Killer/T-Cell Lymphoma: Population-Based Study in the United States
Source: JMIR Form Res. 2025 May 15;9:e70129. doi: 10.2196/70129 (PMC12097651; doi:10.2196/70129)

Table S1. Survival analysis of NKTL patients (N=1162)

| Variables | Univariate | | Multivariate | |
| --- | --- | --- | --- | --- |
|  | HR (95%CI) | *P* | HR (95%CI) | *P* |
| Year of diagnosis |  | 0.001 |  | 0.143 |
| 2001~2005 | Ref. |  | Ref. |  |
| 2006~2010 | 0.862 (0.704-1.057) | 0.154 | 0.935 (0.758-1.154) | 0.531 |
| 2011~2015 | 0.774 (0.631-0.950) | 0.014 | 0.862 (0.653-1.138) | 0.294 |
| 2016~2020 | 0.617 (0.487-0.781) | < 0.001 | 0.714 (0.531-0.959) | 0.025 |
| Age (y) |  | < 0.001 |  | < 0.001 |
| ≤39 | Ref. |  | Ref. |  |
| 40~69 | 1.197 (0.988-1.449) | 0.066 | 1.214 (0.999-1.476) | 0.052 |
| ≥70 | 1.974 (1.589-2.451) | < 0.001 | 1.839 (1.447-2.338) | < 0.001 |
| Median household income |  | 0.047 |  | 0.179 |
| >$70,000 | Ref. |  | Ref. |  |
| $50,000~69,999 | 1.200 (1.030-1.400) | 0.020 | 1.099 (0.939-1.287) | 0.241 |
| <$50,000 | 1.234 (0.910-1.674) | 0.177 | 1.307 (0.957-1.784) | 0.093 |
| First malignant or not |  | < 0.001 |  | 0.083 |
| Yes | Ref. |  | Ref. |  |
| No | 1.534 (1.221-1.919) |  | 1.239 (0.972-1.578) |  |
| Clinical stage |  | < 0.001 |  | < 0.001 |
| Stage I/Localized | Ref. |  | Ref. |  |
| Stage II/Regional | 1.511 (1.223-1.867) | < 0.001 | 1.699 (1.367-2.111) | < 0.001 |
| Stage III or IV/Distant | 2.932 (2.459-3.496) | < 0.001 | 2.365 (1.932-2.896) | < 0.001 |
| Unknown | 1.788 (1.299-2.461) | < 0.001 | 0.994 (0.695-1.421) | 0.974 |
| Nasal type |  | < 0.001 |  | 0.025 |
| No | Ref. |  | Ref. |  |
| Yes | 0.485 (0.414-0.567) |  | 0.815 (0.681-0.975) |  |
| B symptoms |  | < 0.001 |  | 0.018 |
| No | Ref. |  | Ref. |  |
| Yes | 1.619 (1.281-2.046) | < 0.001 | 1.418 (1.112-1.808) | 0.005 |
| Unknown | 1.673 (1.396-2.004) | < 0.001 | 1.176 (0.921-1.501) | 0.193 |
| Treatment |  | < 0.001 |  | < 0.001 |
| No/Unknown | Ref. |  | Ref. |  |
| Only Radia | 0.563 (0.435-0.728) | < 0.001 | 0.623 (0.471-0.824) | 0.001 |
| Only Chemo | 0.808 (0.659-0.989) | 0.039 | 0.707 (0.565-0.887) | 0.003 |
| Radia & Chemo | 0.293 (0.237-0364) | < 0.001 | 0.373 (0.293-0.476) | < 0.001 |

Table S2. Survival analysis of NKTL patients based on different sites of involvement (N=1162)

| Site of involvement | N (%) | Median OS，  m (95%CI) | 1-year OS (%) | 3-year OS (%) |
| --- | --- | --- | --- | --- |
| G1 Nasal cavity | 581 (50.0) | 63.0 (33.8-92.2) | 69.1 | 57.2 |
| G2 paranasal sinus | 88 (7.6) | 13.0 (0-38.1) | 51.1 | 47.2 |
| G3 Oropharynx and nasopharynx, pharynx and throat | 164 (14.1) | 14.0 (6.9-21.1) | 53.1 | 39.0 |
| G4 Gastrointestinal tract | 44 (3.8) | 8.0 (3.7-12.3) | 37.6 | 34.5 |
| G5 Respiratory tract, pleura, and mediastinum | 18 (1.5) | 1.0 (0-2.4) | 16.7 | 16.7 |
| G6 Bone, bone marrow | 10 (0.9) | 4.0 (0-16.4) | 40.0 | 13.3 |
| G7 Lymph nodes, spleen and Waldeyer's ring | 146 (12.6) | 6.0 (4.1-7.9) | 35.0 | 23.3 |
| G8 Skin, soft tissue | 62 (5.3) | 9.0 (4.1-13.9) | 43.5 | 25.8 |
| G9 Others^1^ | 36 (3.1) | 9.0 (3.8-14.2) | 34.3 | 19.0 |
| G10 Unknown^2^ | 13 (1.1) | 5.0 (0-10.9) | 38.5 | 0 |

^1^ Mammary gland, male/female reproductive system, kidney, lacrimal gland, orbit, central nervous system, thyroid gland, adrenal gland.

^2^ Head, face or neck, NOS and Unknown.

Figure S1. OS of patients based on different sites of involvement


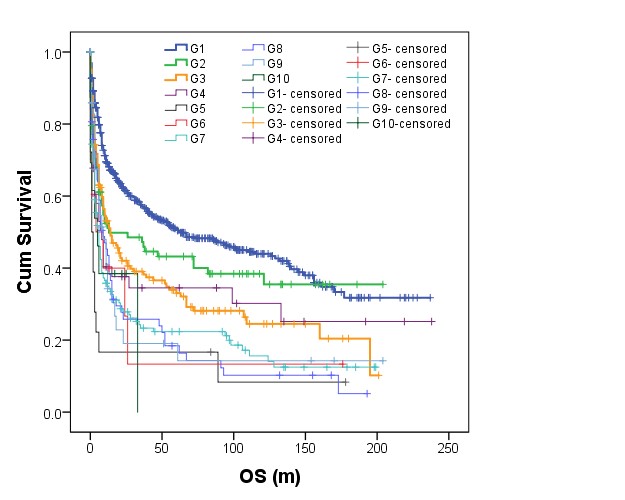


Figure S2. Flow diagram of cohort selection criteria


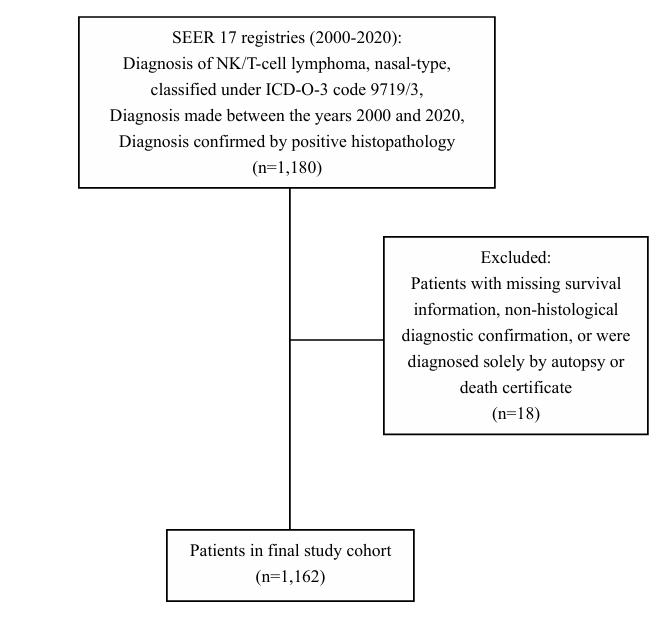

Supplement: Multimedia Appendix 1 [file formative-v9-e70129-s001.docx]
